# Individual-Based Modelling of Bacterial Ecologies and Evolution

**DOI:** 10.1002/cfg.368

**Published:** 2004-02

**Authors:** C. Vlachos, R. Gregory, R. C. Paton, J. R. Saunders, Q. H. Wu

**Affiliations:** 1 BioComputing and Computational Biology Research Group Department of Computer Science University of Liverpool Chadwick Building, Peach Street Liverpool L69 7ZF UK; 2 Department of Electrical Engineering and Electronics Faculty of Engineering University of Liverpool Brownlow Hill Liverpool L69 3GJ UK; 3 Microbiology and Genomics Division School of Biological Sciences University of Liverpool Biosciences Building Liverpool L69 7ZB UK

## Abstract

This paper presents two approaches to the individual-based modelling of bacterial ecologies and evolution using computational tools. The first approach is a fine-grained model that is based on networks of interactivity between computational objects representing genes and proteins. The second approach is a coarser-grained, agent-based model, which is designed to explore the evolvability of adaptive behavioural strategies in artificial bacteria represented by learning classifier systems. The structure and implementation of these computational models is discussed, and some results from simulation experiments are presented. Finally, the potential applications of the proposed models to the solution of real-world computational problems, and their use in improving our understanding of the mechanisms of evolution, are briefly outlined.


**Comparative and Functional Genomics**
*Comp Funct Genom* 2004; **5**: 100–104.
Published online in Wiley InterScience (www.interscience.wiley.com). **DOI:** 10.1002/cfg.368

**Conference Review**

### Individual-based modelling of bacterial ecologies and evolution

C. Vlachos[1], R. Gregory[1,2], R. C. Paton[1]*, J. R. Saunders[3] and Q. H. Wu[2]

[1] *BioComputing and Computational Biology Research Group, Department of Computer Science, University of Liverpool, Chadwick Building, Peach Street, Liverpool L69 7ZF, UK*
[2] *Department of Electrical Engineering and Electronics, Faculty of Engineering, University of Liverpool, Brownlow Hill, Liverpool L69 3GJ, UK*
[3] *Microbiology and Genomics Division, School of Biological Sciences, University of Liverpool, Biosciences Building, Liverpool L69 7ZB, UK*

**Correspondence to:*
*R. C. Paton, BioComputing and Computational Biology Research Group, Department of Computer Science, University of Liverpool, Chadwick Building, Peach Street, Liverpool L69 7ZF, UK.*
*E-mail: R.C.Paton@csc.liv.ac.uk*

Received: 17 November 2003
Revised: 18 November 2003
Accepted: 27 November 2003

### Abstract

**This paper presents two approaches to the individual-based modelling of bacterial ecologies and evolution using computational tools. The first approach is a fine-grained model that is based on networks of interactivity between computational objects representing genes and proteins. The second approach is a coarser-grained, agent-based model, which is designed to explore the evolvability of adaptive behavioural strategies in artificial bacteria represented by learning classifier systems. The structure and implementation of these computational models is discussed, and some results from simulation experiments are presented. Finally, the potential applications of the proposed models to the solution of real-world computational problems, and their use in improving our understanding of the mechanisms of evolution, are briefly outlined. Copyright © 2004 John Wiley & Sons, Ltd.**

**Keywords:** **artificial ecologies; virtual bugs; adaptive behaviour; network-based models; rule-based models; bacterial modelling**

### Introduction

The application of computer science to biology has opened up many new and interesting areas of inquiry. The most obvious has been in the area of genomics and now proteomics. These fields owe their practicality to the ability to store, sort and search massive databases of genetic data with relative ease, giving possibly the only means of amassing and organizing datasets that will grow to be outside of comprehension. Other emerging areas of computation, with regard to modelling and simulation, now allow what is otherwise impossible in the real world, viz. complete measurement (within the limitations of the model resolution) and the ability to restart time and so ask 'what if' questions, knowing that initial conditions really are the same.

A good example of investigating the complex demands of realistic biological models comes from evolution. It is possible to breed bacteria and monitor genetic changes, but measurement is then limited to the population as a whole, rather than the specifics of the individual. Measuring the gene expression levels of a single bacterial cell would mean killing it, ensuring that whatever was learned from that cell can still only be put in the context of the whole population. In the computational world, any parameters, attributes and values are always available. The limitations come from the crude nature of computation as we know it today, compared to the complexity of even the simplest single-celled organism. Fortunately, computers are getting faster and, although we still have decades of improvement ahead to be close to the real world, the flip side of this is that measurement in the real world is likely to not move much further than it is, suggesting that computational biology is, perhaps, the key to understanding how these currently intractable systems evolve.

Copyright © 2004 John Wiley & Sons, Ltd.

### Network-based bacterial modelling — COSMIC

On the basis of the limitations of computational power when compared to the biological counterparts, and the knowledge limitations in real-world organisms, we derived a model now known as COSMIC, which stands for 'computing systems of microbial interactions and communications' [3,4]. This model is based around the biological ideas of evolution and individuality, coupled with the computational requirements of repeatability and, most importantly, tractability. In doing so, we gave this model some important qualities that make it an interesting tool for simulating bacterial evolution that runs almost in real-time, while being entirely quantifiable.

The most central theme of COSMIC is the genome and the proteome. Unlike many simulation-based genomes, the COSMIC genome is organized as a long string of codons over which additional markers denote genes and control sequences (promoters, operators, terminators and attenuators). When transcribed, the gene sequences become the sigma factors, RNA polymerase, repressors, anti-repressors and signalling proteins. It is vital that the gene sequence-to-function mapping be stable, as given a stable interpretation this same genome can be interpreted in the same way in another organism, but it is also vital that the interpretation be fluid enough to allow the incorporation of other gene sequences from other organisms, as well as sequence duplication and deletion events. The ideal solution involves finding the solution to the protein-folding problem but, as this is currently impossible, the only solution is to compare sequence similarity between transcribable genes and control sequences at the codon level.

The overall effect is to fulfil the evolvability requirement. The genome interpretation is stable over the cell's lifetime and carries the same meaning to later generations, while also allowing for a mutating genome — all this without creating semantic problems when combining genomes.

The COSMIC genome and proteome is then encapsulated inside a simulated cell wall. On the cell wall are receptors and flagella that give the cell the ability to sense and move in its environment. In the biological world, the cell acts as the protective bag separating the many proteins and basic chemicals from the outside world. The same is true in COSMIC, as genes and transcription products are all made up of individuals, each with their own lifetime, position in space and set of possible reactants. There can be on the order of 100–1000 genes in a cell's genome, with anywhere from 10 to 500 000 individual proteins in the cell's wall. These are free variables and so can increase further.

The cell presented so far is only one of many, each cell having its own genome and so its own proteome. Each cell coexists in a substrate-enriched environment that is continually changing by the action of the cells themselves. As they consume the substrate, the best areas are continually changing into the worst areas. This leads on to the evolutionary goal of COSMIC, to evolve a genome which uses input from the receptors on the cell wall, to then control the flagella and so move toward the better areas. Growth, and so cell division, depends on the concentration of substrate. Better cells tend to be in better areas of the environment, and so divide more often and replicate their genomes more often, making cell fitness an implicit function.

Figure 1 shows a snapshot of the environment taken during a simulation run. 305 min into the simulation 87 cells are alive in this image, as shown by the black or white dashed circles and numbers (each cell is tracked and recorded individually). A white background shows a nutrient-rich area,

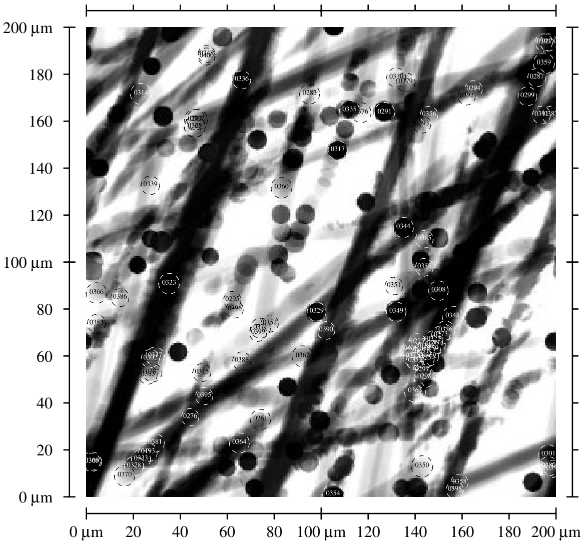

**Figure 1.** Snapshot of the environment taken during a simulation run of the network-based bacterial model (COSMIC)

Copyright © 2004 John Wiley & Sons, Ltd.

black shows a nutrient-restricted area, which also includes dead cells. 218 cells had been tested at this point, all related to different lineages. This simulation then went on to test another 1000 cells before it was stopped with a living population of 412 cells, and having created 95 GB of data to analyse.

The individual-based nature of the simulation means that analysis can be extremely detailed. This, however, comes at a price. COSMIC was designed from the beginning to run on a loosely-coupled computer cluster, thus allowing for the complexity of the model while still making execution almost real-time. With its parallel implementation [4], COSMIC has been placed in a seemingly unique position of having the depth of simulation, the speed of execution and the evolutionary operators (mutation, etc.) to experiment with bacterial evolution in a meaningful way. Current work with COSMIC involves running the system in a Globus II environment.

### Rule-based bacterial modelling — RUBAM

This approach to bacterial modelling is different from the one adopted in COSMIC, in that the environment/organism interactions are modelled using a rule-based approach. The derived system is known as RUBAM, which stands for 'rule-based bacterial modelling'. RUBAM is not intended to be an accurate model of any specific real-world ecologies or bacteria, but rather to capture their most important elements, in order to be capable of exhibiting adaptive and evolvable behavioural patterns. Other models with similar features have been developed in the past, most notably ECHO [6] and HERBY [1].

RUBAM consists of a number of fundamental elements, which work together to construct an artificial ecology. The most important of these are listed below:

- The artificial environment.
- The artificial organisms.
- The environment/organism interaction mechanisms.
- The evolutionary operators.

The artificial environment is represented by an $n$-dimensional grid, in which the artificial organisms are left to survive, interact, multiply and evolve. The environment contains a number of resources in various concentrations, arranged in a specific way that is a function of our problem objectives. The resources provide the 'energy' that is necessary for the organisms to sustain life. The system is designed to conserve matter and energy, which are conflated for the purpose of tractability. Most importantly, the resources (in certain combinations of concentrations) can trigger different types of behaviour in the organisms. This behaviour can be destructive to the organism, or it can generate a desired effect, in which case the organism gradually accumulates enough energy to multiply and generate copies of itself in the environment. In this way, those organisms that are better able to exploit the resources have a higher chance of propagating through to successive generations and maximizing their presence in the population.

The artificial organisms are at the heart of RUBAM, and collectively contain all knowledge gained through evolution. They are implemented using the concept of a learning classifier system (LCS). More details on learning classifier systems and their applications can be found in Holland *et al.* [7] and the references therein. Learning classifier systems are *rule-based* systems (also known as agents or, in this case, virtual bugs), which are able to receive 'messages' coming from external sources (the environment or other organisms present in their neighbourhoods), process them in some way, and generate appropriate actions which can affect the organisms themselves, as well as the outside world. The 'genetic material' of each organism consists of a collection of rules, which map its 'inputs' (known as detectors) to its 'outputs' (known as effectors). This set of rules determines the behaviour of the organism when subjected to different types of stimuli. The objective is to modify the rules in such a way that a desired behaviour is obtained. In the context of evolution, 'desired behaviour' is one that maximizes the lifespan of an individual and guarantees reproductive success.

In order to enable the model to potentially exhibit interesting and complex behavioural patterns, the organisms must be given sufficient degrees of freedom to be able to interact with the environment and themselves. This is achieved by equipping the organisms with a set of detectors and effectors that enable them to modify their energy reserves, be able to sense the presence of resources

Copyright © 2004 John Wiley & Sons, Ltd. 

around them, be able to move in different directions, and also be able to generate signals that can be received by other organisms, thus facilitating coordinated behaviour. The organisms modify their energy reserves by 'consuming' the resources around them. This means that the resource surfaces do not remain static throughout the simulation run, but dynamically change as the organisms continuously interact with the environment. The proposed model was designed to be expandable and customizable, so that additional interaction mechanisms can be introduced as modules to the existing computer code, thus making the model suitable for evolving strategies suited for particular tasks.

Evolution takes place by means of a set of evolutionary operators. These operators are stochastic in nature, and alter the behaviour of the organism in some way by modifying its rules. There are several schemes that could be employed, with mutation (in various forms) being the fundamental evolutionary operator. The behaviour of an organism can also be altered by modifying the 'importance' of each of the rules in the corresponding LCS, based on their observed performance. A popular mechanism for doing this is the bucket-brigade algorithm [5]. Other reinforcement learning algorithms can also be employed. An overview of reinforcement learning and its applications can be found in Kaelbling *et al.* [8].

Another important element in RUBAM is the choice of logic that is used to map the messages received by the detectors to actions taken by the effectors. The proposed system is capable of using both traditional (Aristotelian) logic as well as fuzzy logic [11]. In this work, fuzzy logic was employed because it was experimentally found that it generally resulted in faster convergence to 'well-behaved' rule bases containing fewer rules than using traditional logic. Furthermore, fuzzy logic generally results in rule bases that are easier to comprehend and express in natural language. The inference method used to drive the effectors of the organisms was the one proposed in Mamdani *et al.* [10].

Figure 2 shows a snapshot of a typical RUBAM simulation run, on a two-dimensional grid (i.e. a plane) consisting of $100 \times 100$ points, with an initial population size of 100 bugs. Dark-to-light areas correspond to low-to-high concentrations of resources. Organisms with moderate energy levels are marked with small circles, while those with high energy levels (ready to divide) are marked with large circles. When an organism's energy level drops below a given threshold, then it becomes inactive and does not normally respond to messages. Inactive organisms are marked with crossed circles. In this simulation, the organisms have evolved ways to 'climb' the hills formed by the resources, in order to reach nutrient-rich areas in the environment, which in turn enables them to grow and reproduce. The paths followed by some of them can clearly be observed in Figure 2, all leading to areas of high resource concentrations. Upon examination of the rule bases of the fittest organisms in the population, it was observed that the evolved behaviour closely resembles that of a typical hill-climbing optimization algorithm, viz. the movement towards the direction of ascending gradient of the resource surface. This result, although relatively simple, demonstrates the ability of RUBAM to evolve behavioural patterns that are applicable to real-world problems, such as function optimization problems. Other RUBAM simulation runs have generated strategies that can help locate multiple solutions in multimodal search spaces. Furthermore, RUBAM allows the use of environments of higher dimensions (i.e. $n > 2$), thus enabling the investigation of environments that may not necessarily exist in the natural world, but can be used to solve particular problems.

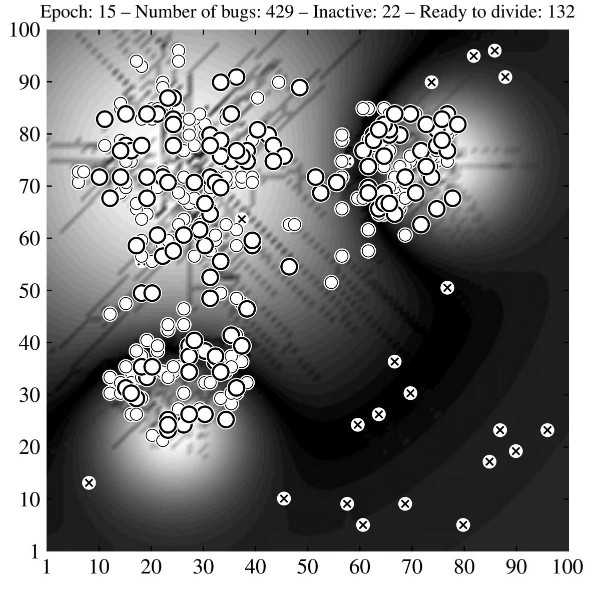

**Figure 2.** Snapshot of the environment taken during a simulation run of the rule-based bacterial model (RUBAM)

Copyright © 2004 John Wiley & Sons, Ltd.

It should be stressed at this point that RUBAM is neither a genetic algorithm (GA) approach [2] nor a genetic programming (GP) approach [9]. There is no external objective function being optimized as such, but rather, a fitness measure that is *internal* to the individual organism. In other words, the fitness of an individual can only be assessed by subjecting it to an environment and letting it interact with it for some time (generally until its energy reserves are exhausted). In contrast to that, in standard GA/GP approaches the fitness of an individual can immediately be computed by means of a known and static objective function. Initial results have shown that RUBAM can generate solutions that are fundamentally (and favourably) different from traditional GA/GP approaches. A disadvantage of the proposed approach, as with most evolutionary approaches, is that it is computationally intensive. The possibility of implementing RUBAM in multiprocessor computer environments is currently being investigated.

### Concluding remarks

The availability of fast computers, large-scale distributed computational resources and other emerging computational tools now allows *in silico* investigations of complex simulations of the evolvability of biological individuals and ecologies. Within this short article, we have discussed two complementary individual-based bacterial modelling systems, which enable questions about microbial evolvability to be investigated, and the evolved knowledge used to help solve real-world problems. Behavioural rules emerging from COSMIC can be tested on the coarser-grained RUBAM system, and questions arising from COSMIC about larger-scale ecological issues can be transferred to RUBAM for further investigations. Initial results have shown that the RUBAM system is capable of evolving optimization algorithms that can be generalized to $n$-dimensional search spaces, and are applicable to real-world optimization problems. It is hoped that further advances in computer technology, particularly in terms of code execution speed and scalability, will result in a wealth of interesting results that can help answer important questions and improve our understanding of the mechanisms of evolution.

### Acknowledgements

The research work reported here has been supported by the EPSRC, e-Science North West/DTI, and MASA.

### References

1. Devine P, Paton RC. 1997. Biologically-inspired computational ecologies: a case study. In *Lecture Notes in Computer Science*, Corne D, Shapiro JL (eds), vol 1305. Springer-Verlag: Berlin; 11–30.
2. Goldberg DE. 1989. *Genetic Algorithms in Search, Optimization, and Machine Learning*. Addison-Wesley: Reading, MA, USA.
3. Gregory R, Paton RC, Saunders JR, Wu QH. 2003. A model of bacterial adaptability based on multiple scales of interaction. In *Computing in Cells and Tissues — Perspectives and Tools of Thought*, Paton RC, Bolouri H, Holcombe M, Parish JH, Tateson R (eds). Springer-Verlag: Berlin (in press).
4. Gregory R, Paton RC, Saunders JR, Wu QH. 2003. Parallelising a model of bacterial interaction and evolution. *BioSystems* (in press).
5. Holland JH. 1985. Properties of the bucket-brigade algorithm. In *Proceedings of the 1st International Conference on Genetic Algorithms and their Applications (ICGA '85)*, Grefenstette JJ (ed.). Erlbaum: Pittsburgh, PA, USA; 1–7.
6. Holland JH. 1992. *Adaptation in Natural and Artificial Systems: An Introductory Analysis with Applications to Biology, Control and Artificial Intelligence*. MIT Press: Cambridge, MA, USA.
7. Holland JH, Booker LB, Colombetti M, *et al.* 2000. What is a learning classifier system? In *Learning Classifier Systems: From Foundations to Applications*, Lanzi PL, Stolzmann W, Wilson SW (eds). Springer-Verlag: Berlin; 3–32.
8. Kaelbling LP, Littman ML, Moore AW. 1996. Reinforcement learning: a survey. *J Artif Intell Res* **4**: 237–285.
9. Koza JR. 1992. *Genetic Programming: On the Programming of Computers by Means of Natural Selection*. MIT Press: Cambridge, MA, USA.
10. Mamdani EH, Assilian S. 1975. An experiment in linguistic synthesis with a fuzzy logic controller. *Int J Man Mach Stud* **7**: 1–13.
11. Zadeh LA. 1965. Fuzzy sets. *Inform Control* **8**: 338–353.

Copyright © 2004 John Wiley & Sons, Ltd. 

